# AI-Assisted Systematic Literature Review of the Economic Burden of Pneumococcal Disease: Development and Validation Study

**DOI:** 10.2196/81049

**Published:** 2026-06-15

**Authors:** Dong Wang, Surabhi Datta, Julie Glasgow, Kyeryoung Lee, Hunki Paek, Jun Zhang, Yi Zheng, Yi-Ling Huang, Long He, Majid Rastegar-Mojarad, Kelsie Cassell, Xiaoyan Wang, Nicole Cossrow

**Affiliations:** 1 Biostatistics and Research Decision Sciences, Merck & Co, Inc, 126 East Lincoln Ave, Rahway, NJ, 07065, United States, 1 732 594 4000; 2 Data Science & Analytics, Intelligent Medical Objects, Rosemont, IL, United States; 3 Clinical Informatics and Terminology Data Engineering, Intelligent Medical Objects, Rosemont, IL, United States; 4 Medical Affairs, MSD R&D (China) Co, Ltd, Beijing, China; 5 Life Sciences, Intelligent Medical Objects, Rosemont, IL, United States; 6 Outcomes Research, Merck & Co, Inc, Rahway, NJ, United States; 7 Department of Health Policy and Management, Tulane University, New Orleans, LA, United States

**Keywords:** pneumococcal disease, economic burden, systematic literature review, natural language processing, generative artificial intelligence, GenAI, artificial intelligence, AI, large language models

## Abstract

**Background:**

Automated systematic literature review (SLR) may reduce the workload and errors associated with manual review, enabling faster, up-to-date reviews even with increasing publication volumes. Large language models (LLMs) have demonstrated strong capabilities in understanding unstructured languages. However, few studies have explored the potential of a comprehensive LLM platform to streamline the entire SLR process from article screening to data extraction.

**Objective:**

This study aimed to investigate the feasibility of applying an LLM-based system to assist with SLR development.

**Methods:**

We developed the Intelligent Systematic Literature Review (ISLaR 2.0) platform, powered by an LLM, and applied it to a use case of the economic burden of pneumococcal disease (PD) literature. First, we established the inclusion and exclusion criteria for the SLR. Second, we defined data elements related to economic burden and domain knowledge, along with guidelines for applying these definitions. Finally, we used the criteria and data element specifications to develop LLM prompts for screening and data extraction. For data extraction, we identified relevant study characteristics and economic burden outcomes. We evaluated ISLaR 2.0’s performance against a gold standard of 50 expert-curated PD articles, using standard metrics (accuracy, precision, recall, and *F*_1_-score). We also conducted a qualitative analysis to describe errors made by the system.

**Results:**

ISLaR 2.0 performed well in abstract and full-text screening (*F*_1_-scores of 86.27 for abstract screening and 87.18 for full-text screening) and data extraction from text (*F*_1_-scores of 92.83 for study details and 79.76 for economic burden outcomes). The *F*_1_-score for data extraction of tabular economic burden outcome data was 94.83. The qualitative analysis revealed 2 main challenges in extracting economic burden details: misclassification of cost categories and failure to extract relevant information.

**Conclusions:**

ISLaR 2.0 enabled efficient execution of an SLR regarding the economic burden of PD. The platform allowed users to flexibly define and modify criteria and data elements, supporting its use across a broad range of health research topics.

## Introduction

Systematic literature reviews (SLRs) in the field of health sciences enable the synthesis of robust and reliable evidence for clinical decision-making and regulatory submissions, as well as the identification of knowledge gaps [1,2]. However, manual SLRs are time consuming and labor intensive [3-5], with one study finding that SLR development typically requires approximately 67 weeks of skilled labor from project initiation to publication [3,6]. Automation can greatly enhance the efficiency of SLR development [4]. To this end, artificial intelligence (AI), including large language models (LLMs), has increasingly been used to assist with SLR activities, ranging from the screening of abstracts [7-9] to full-text data extraction [10-12].

Previous studies of AI-assisted SLR development have often focused either on a single aspect of the process, such as eligible article screening, or on clinical topics, such as treatment efficacy in oncology or immune diseases [7-12]. However, it is important to understand whether AI-assisted SLR tools are suitable for other types of health research, including studies in epidemiology, public health, and health economics. Compared with clinical research, these topics may involve a broader range of biological, social, monetary, and infrastructural factors and thus more varied terminology and outcome measures. Economic burden studies, for example, evaluate the financial impact of a disease on individuals, health care systems, and society, potentially encompassing a wide range of clinical, epidemiological, and monetary measures. SLR development based on conventional AI (eg, supervised machine learning) has been evaluated for economic burden of disease research [13-15]. However, few studies have assessed a comprehensive SLR platform that uses an LLM, specifically GPT-4 (OpenAI), to conduct all stages of an economic burden of disease SLR.

Notably, SLRs of economic burden of disease studies address a critical need to synthesize data to evaluate the wider impact of vaccination programs, including effects on medical costs and health care resource use [16-23]. This data synthesis is relevant for diseases such as pneumococcal disease (PD), a condition associated with a high burden of morbidity and mortality worldwide that may be lowered by pneumococcal vaccines [24-26]. However, conducting SLRs for economic burden of disease and cost-effectiveness research can be challenging. Studies in this area are often lengthy, involve complex and varying methods, include large tables of input variables, and analyze multiple scenarios for a wide range of input values. Thus, data extraction and output consolidation can be time consuming and difficult to standardize across studies.

To address the need for more efficient development of SLRs in economic burden of disease studies, we leveraged the capabilities of generative AI (GenAI) to develop the Intelligent Systematic Literature Review (ISLaR 2.0) system, an LLM-based SLR platform designed to seamlessly conduct SLRs for a broad range of study types. ISLaR 2.0 automates the entire SLR process, from the screening of abstracts and full-text documents to full-text data extraction. This is an enhancement of our ISLaR 1.0 system [15], which can only be used for abstract screening and full-text data extraction. Here, we applied ISLaR 2.0 to a review of the literature on the economic burden of PD in high-risk populations, which served as a use case to evaluate the platform’s capabilities and performance.

## Methods

### Overview

ISLaR 2.0 is a comprehensive SLR platform that incorporates key steps of SLR, including article retrieval from the PubMed and Scopus databases, abstract and full-text screening, data extraction, and results summarization. Thus, ISLaR 2.0 provides LLM-assisted functionality to address the most time-consuming steps of SLR development. For each LLM-assisted step, ISLaR 2.0 uses criteria and other information provided by users through a user interface to construct LLM prompts that guide task processing. Notably, ISLaR 2.0 generates recommendations with explanations for manual human review, thereby incorporating a human-in-the-loop approach into the system. We evaluated the performance of ISLaR 2.0 by comparing the system’s recommendations against a gold standard of 50 manually screened and extracted PD articles. Performance was assessed quantitatively by calculating standard evaluation metrics and qualitatively by manually reviewing articles to describe system errors in screening and data extraction.

### ISLaR 2.0 SLR Workflow

**Figure 1.**
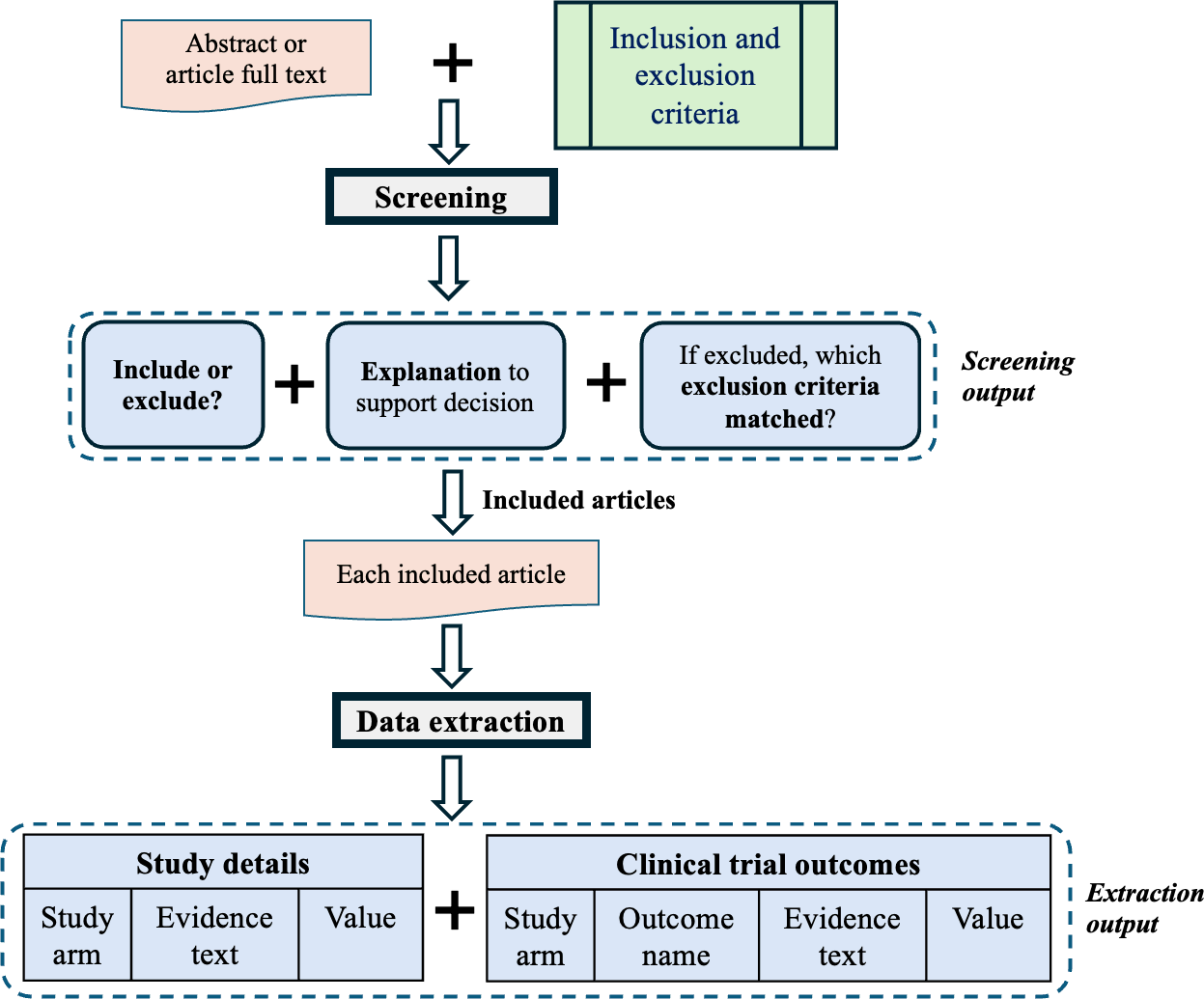
Intelligent Systematic Literature Review (ISLaR 2.0) workflow.

ISLaR 2.0 processes are driven by GPT-4 prompts constructed from information manually input into simple, semistructured user interfaces (Table S1 in Multimedia Appendix 1). These prompts drive the development of the SLR across multiple steps (Figure 1). In step 1, the retrieval of articles from PubMed and Scopus databases is enabled by user input queries derived from topic-specific inclusion and exclusion criteria. In this evaluation, studies of the economic burden of PD were retrieved from the PubMed database using the following query: ((“Pneumococcal disease*“[Text Word] OR “Streptococcus pneumoniae”[Text Word]) AND ((cancer[Text Word]) OR (Immunocompromis*[Text Word]) OR (HIV[Text Word]) OR (“renal disease”[Text Word]) OR (asplenia[Text Word]) OR (diabetes[Text Word]) OR (“heart disease”[Text Word]) OR (“lung disease”[Text Word]) OR (“respiratory disease”[Text Word]) OR (“sickle cell”[Text Word]) OR (“cochlear implant”[Text Word]) OR “cerebrospinal fluid leak”[Text Word])) AND ((cost[Text Word]) OR (“economic burden”[Text Word]))

In step 2, the user inputs the SLR-specific population, intervention, comparison, and outcome (PICO) criteria, which provide the basis for inclusion or exclusion of articles in abstract and full-text screening. The user may specify different criteria for abstracts than for full-text screening, for example, to enable the capture of a broader set of potentially relevant abstracts and a narrower, more accurate set of full-text articles. In this study, we used the same set of criteria at both abstract and full-text screening stages as defined in Table 1, but used different prompts as shown in Table S2 in Multimedia Appendix 1. PICO-based criteria used in this study allowed for a wide range of study designs (eg, clinical trials, real-world evaluations, and meta-analyses), enabling assessment of data extraction for an array of economic burden measures.

**Table 1.** Population, intervention, comparison, and outcome criteria for economic burden studies of pneumococcal disease (PD) studies.

Criteria	Inclusion criteria	Exclusion criteria
Population	Adults (aged ≥18 years) or children (aged 0‐17 years) with PD or a disease associated with *Streptococcus pneumoniae* High-risk populations with PD or diseases associated with *S pneumoniae*, including individuals with cancer, immunocompromising conditions, HIV, renal disease, asplenia, diabetes, heart conditions, lung conditions, sickle cell disease, cochlear implants, and cerebrospinal fluid leaks	Mixed pediatric and adult populations without segregated results Studies that did not report on at least one of the following conditions: pneumococcal pneumonia, nonbacteremic pneumococcal pneumonia, pneumococcal or streptococcal meningitis (including postmeningitis sequelae), acute otitis media, pneumococcal bacteremia (sepsis or septicemia), streptococcal septicemia, all-cause pneumonia^a^, community-acquired pneumonia^a^, unspecified bacterial pneumonia^a^, complicated pneumonia^a^, bacteremic pneumonia^a^, all-cause otitis media (OM)^a^, acute suppurative OM^a^, recurrent OM^a^, complicated OM^a^, OM with tympanostomy tube replacement^a^, empyema^a^, and pleural effusion^a^
Interventions or comparators	Data that are not specific to any therapy Data specific to PD vaccination	—^b^
Outcomes	Study results including at least one of the following economic burden outcomes: direct costs, indirect costs, societal costs, and resource use	Studies not reporting on at least one of the outcomes listed in the inclusion criteria
Study types	Meta-analysis study Review study Original research article study	—
Other	Studies must report data from one or more of the following countries: the United States, Canada, South Korea, South Africa, Japan, Australia, France, Germany, Italy, Spain, Brazil, and member countries of the United Kingdom English language only	Studies in a language other than English Duplicate reports (eg, conference abstracts that report on data that is subsequently published)

a Even if pneumococcal disease was not confirmed, studies reporting at least one of these outcomes were considered eligible.

b Not applicable.

**Figure 2.**
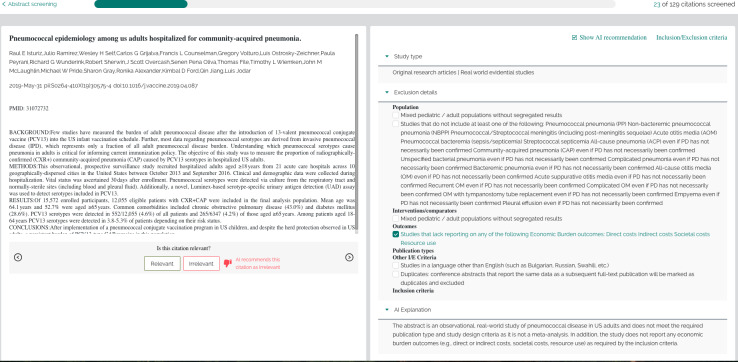
System interface showing large language model–recommended screening decisions.

In step 3, abstract screening is performed using the GPT-4 Turbo model based on manual input of PICO-based criteria, the title, abstract text, additional domain information, and general screening instructions. In this evaluation, screening instructions were designed to be inclusive to favor a high recall of potentially relevant abstracts, minimizing erroneous exclusions during this initial screening. After processing the prompt instructions, ISLaR 2.0 outputs its decision to include or exclude the abstract and, consistent with the system’s human-in-the-loop approach, generates a list of reasons for excluding abstracts, enabling user acceptance or rejection of the system recommendation (Figure 2).

Step 4 entails full-text screening, in which PICO-based criteria, additional domain information, general screening instructions, and the full text of the article are input into the system prompt and processed via the GPT-4 Turbo model. In this study, we used the Amazon Textract tool [21] to convert full-text publications from PDF into text format, facilitating their input into the system prompts for screening and data extraction. To ensure high precision at this stage, we also used stricter screening instructions than those used for abstract screening, namely, all inclusion criteria had to match, and none of the exclusion criteria were allowed to match (Table S2 in Multimedia Appendix 1).

**Figure 3.**
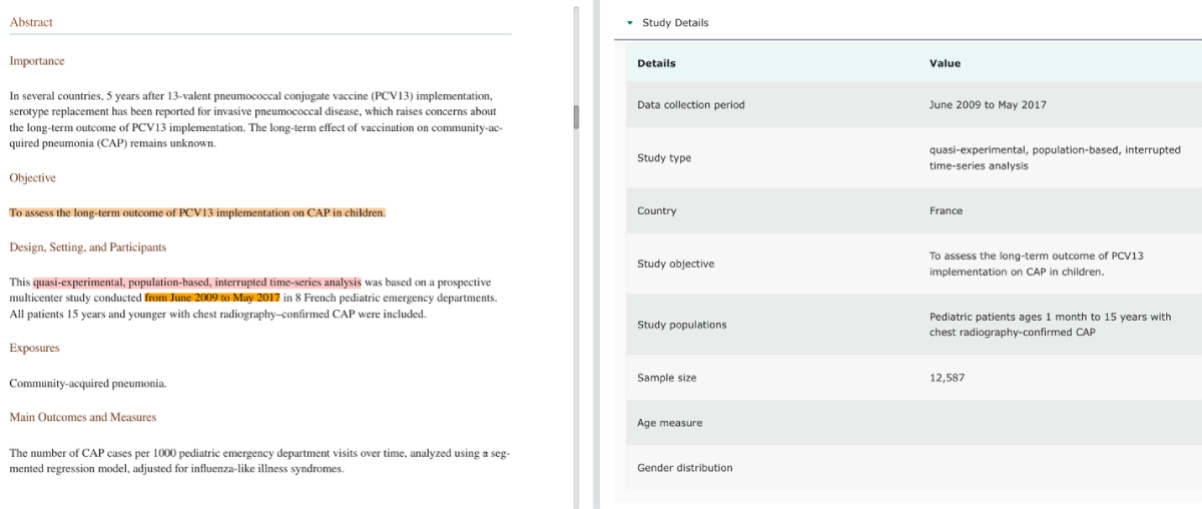
System interface showing large language model–extracted data elements and values evidenced by the highlighted text spans in an article.

Step 5 entails the extraction of user-defined data elements from articles marked for inclusion after full-text screening (Table 2). These data elements and their descriptions are input into the GPT-4 Turbo model prompts in 2 formats, one for the main text of the articles and the other for tabular data. Extracting data from full-text articles involves 2 sets of prompts, one for study details and the other for study outcomes. The study details prompt extracts data relevant to the overall study, including the data collection period, study type, country or continent, study objectives, study populations, sample size, age (number), age unit (eg, years or months), and gender distribution (Figure 3). For each element of the study details prompt, the prompt needs to identify 3 attributes: the element name, its value, and the evidence text span from the full-text article. The study outcomes prompt extracts economic burden outcomes (ie, direct costs, indirect costs, societal costs, and health care resource use) specific to each study cohort. Thus, the study outcomes prompt requires identification of 4 attributes: study cohort, element, value, and the evidence text span from the full-text article. For instance, in an article included in this study the element “Resource use” had an extracted value of “652 cases reduced; 23 deaths reduced; NNV=194” with the associated study cohort of “US black population cohort”; the evidence text span was identified as: “In cohorts of 50-year-olds over their remaining lifetime, the strategies with the greatest public health impact, compared with no vaccination when PPSV was assumed to be ineffective against NBP, reduced PD cases by 652 (number needed to vaccinate [NNV] to prevent 1 case=194) and deaths by 23 among a cohort of 549,197 blacks.”

**Table 2.** Description of data elements.

Element category and study detail	Description
Data collection period	The time frame of data collection
Study type	The type of study design (eg, randomized controlled trial study, observational study, cross-sectional study, database claims analysis, and cost-effectiveness study)
Country or continent	The country and/or the continent associated with the study
Study objective	Overview of study details, such as the objective outlined in the article
Study populations	Population with pneumococcal disease and possible high-risk conditions (eg, pneumococcal disease without high risk, cancer, immunocompromising conditions, HIV, renal disease, asplenia, diabetes, heart conditions, lung conditions, sickle cell disease, cochlear implants, and cerebrospinal fluid leaks)
Sample size	The number of participants included in the study
Age measure	The measure used to report for age (eg, mean and median)
Age unit	The unit of measure used to report age (eg, years)
Gender distribution	The gender distribution of study participants
Direct costs	The cost of direct patient care for pneumococcal disease, including the units of measure (eg, “mean cost per year,” “median cost per patient,” and “total cost for a cohort”)
Indirect costs	Short- and long-term lost productivity associated with pneumococcal disease
Societal costs	The total cost to a society resulting from pneumococcal disease (eg, mortality-related costs)
Resource use	Health care resource use related to pneumococcal disease, including care costs, numbers of inpatient and outpatient visits, inpatient length of stay, percent reductions in inpatient visits, the number of medical tests and procedures, and specification of the associated time of health care resource use

To facilitate generation of input data for the study outcomes prompt, study cohorts are first identified before constructing the prompt using a separate prompt with the following instructions:

Extract detailed names or descriptions of all cohorts, sub-cohorts, sub-groups, and study arms mentioned in the following article.

Subsequently, cohort specifications are input into the outcome extraction prompt with the following instructions:

We aim to extract all relevant information related to economic burden from the following article (full text). The following are some information categories or data elements we are interested in: <list of all data elements with their descriptions followed by detailed instructions and article full text>.

In addition to the attributes defined for each data element, additional domain knowledge may be input into data extraction prompts. During data extraction, the system highlights the relevant section of text and the extracted value and allows the user to directly edit any extracted values, in case errors have occurred (Figure 3).

In this study, data extraction prompts were developed and optimized using 5 articles on the economic burden of PD randomly selected from those identified in the PubMed database search (step 1). These 5 studies were not among those included in the performance evaluations, as described in the next section. Prompts for extracting economic burden outcomes were initially constructed in ISLaR 2.0 using the GPT-4 Turbo model. However, the GPT-4 Turbo model showed suboptimal performance for extracting tabular data due to errors in automatically converting long and multiheader tables from PDFs into text format. To address this issue, the GPT-4o model was tested for its image-processing capabilities, wherein each table was fed into the model as an image and prompted to extract and organize economic cost information. Information identified in tables was organized by attributes such as study cohort, element, or value.

### Evaluation

To evaluate the system performance, we selected 50 expert-curated articles from the pool of 108 retrieved articles. A gold standard was established through expert manual review following a focused calibration period using articles not included in the study pool. Calibration used a scope-boxed collaborative approach and continued until all experts agreed that bias had been substantially mitigated and screening decisions were aligned to ensure consistency. A senior physician with prior SLR experience then independently screened all abstracts and included full-text articles. For cases in which inclusion or exclusion decisions were ambiguous, consensus discussions were conducted among a multidisciplinary team comprising one physician and 3 PhD-level researchers to resolve disagreements and minimize potential bias. For abstract screening, the evaluation dataset consisted of 50 expert-curated articles, which served as the gold standard. For full-text screening, the evaluation dataset consisted of 23 articles labeled as “relevant” in human screening of the abstracts of the original 50 selected articles. Data extraction was evaluated using 19 articles labeled as “relevant” in human screening of full-text articles. We calculated performance scores by comparing the system predictions against the gold standard. Screening performance was based on comparisons between the final screening decision to include or exclude the article and the corresponding gold standard classification. Data extraction performance was based on a comparison of system-extracted data elements and associated study cohort information with those of the gold standard. Quantitative evaluations of performance were conducted by calculating 4 standard evaluation metrics: accuracy, precision, recall, and *F*_1_-scores. Accuracy was defined as the percentage of correct predictions made by the system out of the total number of predicted instances. Precision was defined as the percentage of correctly classified positives out of all positives predicted. Recall was defined as the percentage of correctly classified positives out of all actual positives. *F*_1_-scores were calculated as the harmonic mean of precision and recall, providing a comprehensive assessment of the system performance. We also conducted a qualitative analysis of the errors made by the system to identify challenging areas in screening and data extraction. All annotation and evaluation procedures were conducted using fully blinded protocols, with annotators kept independent of the system development process to minimize potential bias.

## Results

### Screening Performance

The system performance for abstract and full-text screening is shown in Table 3. The system achieved a recall of 95.65% for abstract screening and 89.47% for full-text screening. The *F*_1_-score was 86.27% for abstract screening and 87.18% for full-text screening. Screening classification results are summarized in the confusion matrices in Table 4.

**Table 3.** Performance metrics for abstract and full-text screening.

Module	Accuracy (%; 95% CI)	Precision (%; 95% CI)	Recall (%; 95% CI)	*F*_1_-score (%)
Abstract screening (n=50)	86.00 (74–93)	78.57 (60–90)	95.65 (79–99)	86.27
Full-text screening (n=23)	78.26 (58-90)	85.00 (64-95)	89.47 (69-97)	87.18

**Table 4.** Confusion matrices for abstract and full-text screening.

	System prediction: relevant	System prediction: irrelevant	System prediction: total
Abstracts screened, n			
Gold standard: relevant	22	1	23
Gold standard: irrelevant	6	21	27
Gold standard: total	28	22	50
Full texts screened, n			
Gold standard: relevant	17	2	19
Gold standard: irrelevant	3	1	4
Gold standard: total	20	3	23

### Data Extraction Performance

Among the 19 (100%) articles labeled as relevant in the full-text gold standard screening, 13 (68.4%) contained information on economic costs and resource use in their tables, and 6 articles (31.6%) either did not contain any tables or contained tables without relevant information. Thus, all 19 (100%) articles were used to evaluate data extraction from the main text, while 13 (68.4%) articles containing a total of 20 tables were used to evaluate extraction of tabular data.

Table 5 shows the system performance in extracting data elements. *F*_1_-scores for GPT-4 Turbo–based data extraction from the main text were 92.83% for identifying the study details and 79.76% for identifying economic burden elements. The *F*_1_-score for GPT-4o–based extraction of economic burden elements from tables was 94.83%. Furthermore, for the main text, we calculated the performance scores for individual attributes (ie, the associated cohort, the element, and the value) of the economic burden elements. The *F*_1_-scores were 92.55% for identifying the study cohort, 80.00% for identifying the data element, and 84.88% for identifying the value (data not shown in the table).

**Table 5.** Performance measures for data element extraction from the full text of 19 articles.

Data element category	Accuracy (%; 95% CI)	Precision (%; 95% CI)	Recall (%; 95% CI)	*F*_1_-score (%)
Study details from main text (GPT-4 Turbo)	87.15 (82-91)	87.13 (81-91)	99.33 (96-100)	92.83
Economic burden outcomes from tables (GPT-4o)	90.17 (87-92)	90.67 (88-93)	99.39 (98-100)	94.83
Economic burden outcomes from main text (GPT-4 Turbo)	66.34 (57-75)	74.44 (65-82)	85.90 (77-92)	79.76

### Error Analysis

A manual review of system errors in screening identified reasons for false positive and false negative errors. False positive errors in abstract screening occurred when the system incorrectly included articles focused on clinical outcomes rather than economic burden outcomes. False positive errors in full-text screening occurred when the system misidentified criteria for high-risk populations and age groups. For example, the system included an article regarding nonimmunocompromised older adults, which was not a high-risk population as defined in the PICO criteria (Table 1). False negative errors in full-text screening occurred when the system excluded articles based on an incorrect interpretation of the study design criteria (eg, the system excluded a meta-analysis study).

In total, 22 errors were made during the system extraction of study detail data from the main text of the 19 articles (Table 6). These errors involved misinterpretation of the study type, failure to identify age units, and incomplete extraction of information on the data collection period and sample size. A total of 23 false positive errors and 11 false negative errors occurred (Table 7) with the GPT-4 Turbo–based extraction of economic burden data from the main text of 19 articles. False positive errors involved misclassifying the burden elements, inaccurately capturing burden values, and failing to identify some indirect costs. One false positive error was linked to both element misclassification and inaccurate cohort identification. The GPT-4o model performed better than the GPT-4 Turbo model in extracting tabular economic burden data. Two false positive errors and 3 false negatives were observed (not shown in the table) when using GPT-4o to extract economic burden data from 20 tables in 19 articles. The false positive errors occurred in 2 tables, from which cohort information was partially captured (eg, missing additional information such as “initial CD4 count of 350 cells/mm^3^” and “100,000 HIV-infected 30-year-old patients”). Furthermore, in one table, an element description was missing an important detail (“related to influenza vaccination”). All false negative errors in GPT-4o–based data extraction were associated with the system being unable to identify the value in the first cell of the first row of the table.

**Table 6.** The top 3 incorrectly extracted study detail data elements (n=22).

Element name	Error prevalence, n (%)	Example
Study type	6 (27.3)	The system predicted a real-world data analysis study to be an observational study [27]
Age unit	5 (22.7)	The system failed to identify “years” for a study involving younger and older adults [28]
Data collection period and sample size	3 (13.6)	Data collection period: the system extracted “January 1, 2000, to July 5, 2010,” which was partially correct but missed an important detail about search update: “The search was updated monthly through the AutoAlert function of the search up to January 31, 2011” Sample size: the system identified “100,000”; however, the complete information should have been “a hypothetical cohort of 100,000 50-year-old adults [29]”

**Table 7.** Errors in GPT-4 Turbo–based extraction of economic burden data from the main text.

Error categories	Error prevalence, n (%)	Example
False positive errors^a^ (n=23)		
Elements misclassified	11 (47.8)	The system mistakenly labeled the societal costs as direct costs for the article text stating “...a single dose PCV13 strategy costs $70,937 per quality adjusted life year (QALY) gained compared to no vaccination [30]”
Economic burden values not extracted meaningfully	5 (21.7)	The system extracted “$13.9 million for at-risk persons” as direct costs associated with annual cost of all-cause pneumonia; however, it missed an important detail “per 100,000 persons” [31]
Element lacked context	3 (13)	The system extracted “Direct costs” instead of “Direct costs - mean cost of hospital stay for ICU-admitted patients” [32]
Extraneous elements identified	3 (13)	The system extracted “Costs of vaccine, program development, and side effect treatment” as a direct cost, although this information was not requested [33]
Study cohort identified inaccurately	2 (8.7)	The system predicted “Entire cohort” instead of “Vaccinated cohort” for a societal cost element [34]
False negative errors (n=11)		
Indirect costs	7 (63.6)	Vaccination cost and incremental cost-effectiveness ratio (ICER) [35]
Direct costs	2 (18.2)	Total cost of vaccination plus treating pneumonia [34]
Societal costs	1 (9.1)	Total projected cost of treating pneumonia [34]
Resource use	1 (9.1)	*Streptococcus pneumoniae* was associated with the largest burden for adults [36]

a The numerators for false positive errors add up to 23 because 1 error involved both element misclassification and inaccurate cohort identification.

## Discussion

### Principal Findings

We developed an LLM-based intelligent SLR platform, ISLaR 2.0, and evaluated the platform’s performance in completing complex tasks involved in developing SLRs for economic burden studies. While previous research has primarily focused on specific aspects of the SLR process, such as eligible article screening [8-10], PICO element extraction [37], or data extraction in small studies as a proof of concept [11], our ISLaR 2.0 platform automated the entire SLR workflow. This included comprehensive screening of eligible articles based on abstracts and full-text review, as well as extraction of data from full-text articles.

Several existing SLR automation tools have been developed [38], including Trialstreamer [39], SWIFT-Review [40], DistillerSR [41], NestedKnowledge [42], SWIFT-ActiveScreener [43], Abstrackr [44], EPPI-Reviewer [45], and RobotReviewer [46]. Tools such as Trialstreamer and SWIFT-Review primarily focus on expediting the scoping process prior to initiating a living systematic review by identifying studies most likely to be relevant or of higher quality, often with minimal user input. DistillerSR, NestedKnowledge, Abstrackr, and EPPI-Reviewer use active learning to screen and reorder references, thereby prioritizing those likely to be relevant for human review. SWIFT-ActiveScreener further assists reviewers by estimating screening completeness and notifying them when manual screening may be stopped early. With respect to data extraction, relatively few tools have automated this component. RobotReviewer and Trialstreamer, for example, implement semiautomated methods for extracting data from eligible articles. In contrast, our study introduces an end-to-end AI-assisted SLR pipeline that enables both abstract and full-text screening with supporting explanations, as well as comprehensive data extraction, all guided by user feedback and preferences. The human-in-the-loop interface provides a comprehensive view of each abstract, along with an AI-recommended disposition and supporting rationale. The rationale includes details for each relevant exclusion criterion, as well as an overarching AI explanation. This approach gives human users maximum control over inclusion and exclusion decisions while streamlining the assessment and disposition process.

Wang et al [12] made strides in accelerating the SLR process by establishing an LLM-based pipeline focused on oncology therapy studies. In this study, we developed a generalizable LLM-assisted platform that can be easily adapted to different topics and outcome data elements. To evaluate the effectiveness of ISLaR 2.0, we applied it to a use case involving the literature on the economic burden of PD, allowing assessment of the platform’s capabilities regarding health economic studies. Our experience suggests that ISLaR 2.0 has the potential to enable a small team of researchers to perform all steps of an SLR in a shorter time frame than a conventional SLR, although the speed and efficiency of the system should be formally evaluated in further studies.

Findings of this study showed that ISLaR 2.0 had high recall during abstract screening and high precision during full-text screening (>85%), indicating its robustness at both stages in identifying eligible articles. The system also exhibited strong performance in extracting information on study characteristics and capturing important aspects of economic burden outcomes. While developing ISLaR 2.0, we noted challenges in extracting tabular data using the GPT-4 Turbo model. Although the system’s GPT-4 Turbo prompts worked well for extracting numeric cost values associated with the burden outcomes, they often lacked accuracy in extracting the appropriate study cohort and units in the burden element values. This could have been due to the complex and varied structure of the tables or difficulties in converting tables from PDF documents to text. Our experiments with the GPT-4o model using table images yielded a substantially improved performance, including an evaluation *F*_1_-score of 94.83%, demonstrating the system’s capability to capture most of the relevant tabular data. Further research should be conducted to examine the scalability of this approach.

In the future, we aim to further improve the ISLaR system by evaluating prompt refinement to enable more accurate extraction of study detail elements and outcomes. For economic burden studies, this could include providing a more precise interpretation of societal costs, adding descriptions of how cost categories differ from one another, and providing example scenarios in the prompts. Accuracy during data extraction may also be improved by defining and incorporating examples of common concepts related to each cost category. Notably, ISLaR 2.0 does not extract information from supplementary data in articles, which often include relevant economic burden–related details. Thus, we intend to expand the system’s capacity to include a review of supplementary data. In addition, while prior work has demonstrated substantial time savings associated with the ISLaR platform [47], this study did not formally quantify reviewer time-on-task or implementation costs, which we hope to examine further in future evaluations. The inclusion criteria may also have biased cost estimates toward high-income settings, potentially limiting the generalizability of the findings to lower- and middle-income countries. Similarly, this study was conducted using a limited corpus within a defined disease area, which may restrict the generalizability of the findings. Although appropriate for a feasibility assessment, future research should evaluate the performance of the proposed workflow across larger and more diverse datasets spanning multiple disease domains. Additionally, further investigation may be warranted to determine whether hardware-specific optimizations influence the performance or efficiency of locally deployed LLMs used for systematic literature screening. Looking ahead, we aim to examine the applicability of this SLR system to other topics (eg, clinical trials, public health, and epidemiology) to better assess its generalizability.

### Conclusions

This study demonstrated how the capabilities of an advanced LLM can be harnessed to conduct rigorous SLR tasks involved in the analysis of the economic burden of PD. With appropriate human-in-the-loop review and oversight, ISLaR 2.0 may substantially reduce human resource requirements for SLR development by decreasing time and cost. Moreover, by providing an end-to-end solution for SLR development that accurately identifies and extracts relevant information from a wide range of study types, our AI system may accelerate evidence generation for the economic burden of diseases and other research areas.

## Supplementary material

10.2196/81049 Multimedia Appendix 1 Prompt structure, templates, and instructions used in the large language model prompt to guide the screening processes.

## Abbreviations

AI: artificial intelligence
ISLaR 2.0: Intelligent Systematic Literature Review
LLM: large language model
PD: pneumococcal disease
PICO: population, intervention, comparison, and outcome
SLR: systematic literature review
