# Supplementary material for: AI-Assisted Systematic Literature Review of the Economic Burden of Pneumococcal Disease: Development and Validation Study
Source: JMIR AI. 2026 Jun 15;5:e81049. doi: 10.2196/81049 (PMC13268435; doi:10.2196/81049)
Supplement: Multimedia Appendix 1 [file ai-v5-e81049-s001.docx]

**Table S1.** Prompt structure and templates

| **Abstract screening prompt ^a^** |
| --- |
| Article abstract screening: Identify if the following abstract is eligible given the following inclusion and exclusion criteria guidelines for the disease -- <disease_name>.  Use the following contextual information to process the meaning of terms and abbreviations in the criteria and abstract text:  <additional_domain_knowledge>  The following are the inclusion and exclusion criteria that should be followed for screening:  [INCLUSION CRITERIA FOR PUBLICATION TYPE]:  [INCLUSION CRITERIA FOR STUDY DESIGN]:  [INCLUSION CRITERIA]:  <inclusion_criteria>  [EXCLUSION CRITERIA]:  <exclusion_criteria>  [ABSTRACT TITLE]: <title_of_article>  [ABSTRACT TEXT]: <abstract_of_article>  Based on the above abstract title, abstract text, and the inclusion/exclusion criteria, answer either "Include" or "Exclude" under "Screening decision".  Please strictly follow the conditions in the inclusion and exclusion criteria points for taking the eligibility decision.  Besides the main screening decision, provide responses for the following:   1. Provide explanations of the precise reasoning for the eligibility decision under "Note/Explanation". 2. In case of "Exclude" decision, show the specific exclusion   Return the results strictly in the following format:  The output should be formatted as a JSON instance |
| **Data extraction prompt** |
| 1. Study details extraction prompt   *Extract information for the elements below from the following article full text:*  *<data_element_name>: <data_element_description>*  *<additional_domain_knowledge>*  *[ARTICLE TITLE]: <title_of_article>*  *[ARTICLE FULL TEXT]: <full_text_of_article>*  *The output should be formatted as a JSON instance.*   1. Economic burden outcomes extraction prompt   This is done in steps. First, study cohorts are identified from the full-text of an article. These cohorts are then used to extract economic burden outcome details both from the main text and the article tables.   - 1. Study cohort identification prompt   Instructions to Extract Detailed Information on Cohorts, Sub-Cohorts, Sub-Groups, and Study Arms  1) Extraction Objective:  Extract detailed names or descriptions of all cohorts, sub-cohorts, sub-groups, and study arms mentioned in the following article.  2) Clarity in Presentation:  Specify each cohort in a clear and concise manner to facilitate easy identification and understanding. Aim for clarity and accuracy in describing each cohort to avoid ambiguity.  3) Task Execution:  Ensure comprehensive coverage by extracting all mentioned names or descriptions related to cohorts, sub-cohorts, sub-groups, and study arms.  [ARTICLE TITLE]: <title_of_article>  [ARTICLE FULL TEXT]: <full_text_of_article>  - List the full details of all cohorts, sub-cohorts, sub-groups, and study arms mentioned in the article. Use commas to separate each entry.   - 1. Economic burden outcomes extraction from main text of an article   We aim to extract all relevant information related to cohort from the following article full-text. The following are some information categories or data elements we are interested in:  * <data_element_name>: <data_element_description>  Study Cohorts in the Article: <cohorts_extracted_by_previous_prompt>  Extraction Steps:   1. Review the Provided Article: 2. Task Objective:   - Extract and organize relevant information such as data elements, results, findings   1. Output Format:   - Present extracted information in a structured computational format.  Use the following contextual information to interpret the meaning of terms and abbreviations in the instructions and article text. Also follow any specific instructions if provided. Also follow any specific instructions if provided:  <additional_domain_knowledge>  [ARTICLE TITLE]: <title_of_article>  [ARTICLE FULL TEXT]: <full_text_of_article>  Please ensure to populate the final output accurately with the extracted data and use appropriate formatting to ensure clarity and readability.  The output should be formatted as a JSON instance that conforms to the JSON schema below.  As an example, for the schema {"properties": {"foo": {"title": "Foo", "description": "a list of strings", "type": "array", "items": {"type": "string"}}}, "required": ["foo"]}  the object {"foo": ["bar", "baz"]} is a well-formatted instance of the schema. The object {"properties": {"foo": ["bar", "baz"]}} is not well-formatted.  Here is the output schema:  ```  {"properties": {"entries": {"title": "Entries", "type": "array", "items": {"$ref": "#/definitions/Element"}}}, "required": ["entries"], "definitions": {"Element": {"title": "Element", "type": "object", "properties": {"study_cohort": {"title": "Study Cohort", "type": "string"}, "name": {"title": "Name", "type": "string"}, "description": {"title": "Description", "type": "string"}, "text_span": {"title": "Text Span", "type": "string"}, "value": {"title": "Value", "type": "string"}}, "required": ["name", "description"]}}}  ``` |

^a^ The abstract- and full-text-screening prompts follow a consistent structure and methodology, with minor adaptations in the full-text prompt to incorporate additional context available from complete articles.

**Table S2.** Instructions used in the LLM prompt to guide the screening processes

| **Abstract screening** |
| --- |
| To help make the eligibility decision, please carefully consider the following instruction:  Does any one of the exclusion criteria under Population, Intervention/Comparators, Outcomes, or Other categories perfectly match given the abstract title and text? If the answer is clearly "Yes", then "Exclude" the abstract, otherwise "Include" the abstract.  Note: If there is insufficient information in the abstract to decide whether to include or exclude the abstract, please consider to "Include" the abstract. |
| **Full-text screening** |
| To help make the eligibility decision, please carefully consider the following steps one by one:   1. The article's study design and publication type value are – [study_design_publication_type_extracted_by_separate_prompt]. Does any one of the exclusion criteria under Population, Intervention/Comparators, Outcomes, or Other categories perfectly match given the article's full text and the article's study design and publication types? 2. If answer to question 1 is clearly "Yes", only then "Exclude" the article, otherwise, consider the following five questions related to inclusion criteria:    1. Does each and every inclusion criterion under Population category strictly match given the article?    2. Does each and every inclusion criterion under Intervention/Comparators category strictly match given the article?    3. Does each and every inclusion criterion under Outcomes category strictly match given the article?    4. The article's study design and publication type value are – [study_design_publication_type_extracted_by_previous_prompt]. Only in case any study design or publication type criteria is mentioned under the inclusion criteria, consider this question, otherwise please do not consider this question. The question is - Does the article's study design and publication type value belong to any one of the study design and publication type categories mentioned under the inclusion criteria?    5. Does each and every inclusion criterion under other category strictly match given the article? 3. If answer to question 2.4 is "No", then "Exclude" the article. Otherwise, if answers to all five questions above under step 2 (that is, 2.1, 2.2, 2.3, 2.4, and 2.5) are clearly "Yes", then "Include" the article.   For all other scenarios, label as "Include - (not very confident)".  Note: Classify an article as "Include - (not very confident)" only when you are not very confident about including an article based on the information in the inclusion and exclusion criteria points. |
